# Choanal atresia, analysis of 16 cases - the experience of HRAC-USP from 2000 to 2004

**DOI:** 10.1016/S1808-8694(15)31240-4

**Published:** 2015-10-20

**Authors:** Gilberto da Fontoura Rey Bergonse, Arakem Fernando Carneiro, Trissia Maria Farah Vassoler

**Affiliations:** Resident physician in Otorhinolaryngology, Hospital de Reabilitação de Anomalias Craniofaciais - USP/Bauru; Preceptor and Head, Department of Otorhinolaryngology, Hospital de Reabilitação de Anomalias Craniofaciais - USP/Bauru; Resident physician, Otorhinolaryngology, Hospital de Reabilitação de Anomalias Craniofaciais - USP. Hospital de Reabilitação de Anomalias Craniofaciais - USP

**Keywords:** choanal atresia

## Abstract

Choanal atresia is a rare disease and the incidence is 1: 5000-8000 newborns alive. It is more comom among female and usually is associated with others malformations.

**Study design:**

clinical retrospective. When unilateral its diagnose may be postpone because its symptoms are less life threating. But when bilateral children have important respiratory insuficency. This articles will report incidence and the expirience of HRAC-USP.

## INTRODUCTION

Congenital choanal atresia is an uncommon disease, reported and described by Roederer in 1755. In 1830, Otto recognized this condition as an anatomical disorder and the first surgery was performed by Emmert in 18511., 2., 3..

It shows an incidence of 1:5,000-8,000 of live newborns, among which females are more affected than males in a 2:1 ratio. Unilateral defects are more common than bilateral ones, while the right nostril is twice more affected than the left nostril^4^^,^^5^.

Presumably, 90% of defects are of bony type, although recent literature shows that 70% of cases are mixed defects, that is, of bone-membranous type1., 2., 3..

Association with other anomalies and syndromes ranges from 20 to 50%, with no genetic corroboration; the most common syndrome is CHARGE (coloboma, cardiovascular malformations, growth and mental retardation, urogenital and ear anomalies^1^^,^^4^^,^^5^).

There are several theories explaining choanal atresia: I – persistence of oral-pharyngeal membrane; II – failure of the physiological oral-nasal membrane rupture; III – mesodermic tissue adherence; IV – growth of palatal processes1., 2., 3.^,^^6^.

Diagnosis is dependent on high suspicion level. Bilateral atresia presents nasal obstruction, stridor, cyclic cyanosis (improving with newborn's cry). Unilateral atresia may not be perceived for many years and follows with late rhinorrhea and unilateral nasal congestion; however, some patients may remain oligosymptomatic^6^.

Complementary tests, such as computerized tomography of facial sinuses and nasofibroscopy, are essential for diagnosis and therapeutic plan - involvement, localization, and distinctive bone, membrane and mixed atresias^7^.

Initial treatment consists of oral airway maintenance (orotracheal intubation, if required) up to definite surgical correction, which must recover nasal airflow, avoid facial growth damages by means of a safe and fast technique. Today, several types are performed: microscopic and endoscopic transnasal, transpalatal, transseptal, transantral techniques and intranasal dilatations^2^^,^^6^^,^^8^^,^^9^.

This article presents a review of our experience at the Craniofacial Anomalies Rehabilitation Hospital of Universidade de São Paulo in the period of 2000 to 2004.

## MATERIAL AND METHODS

Retrospective study based on medical record review of the Craniofacial Anomalies Rehabilitation Hospital of Universidade de São Paulo in the period of 2000 to 2004. The patients included in the study were those submitted to corrective surgery of choanal atresia, assessed according to time of diagnosis and treatment, sex, associated anomalies, complications, permanence of stent, need of surgical revision, time of follow-up, uni or bilateral affection, as well as existence of choanal stenosis only and surgical technique adopted.

## RESULTS

Eighteen medical records were selected, out of which 2 were excluded due to lack of data. Sixteen patients (12 females and 4 males), ages varying from newborns up to 20 years old, received treatment at the Craniofacial Anomalies Rehabilitation Hospital of Universidade de São Paulo – Bauru in the period of 2000 and 2004.

Unilateral choanal atresia was observed in 50% (8) of the patients and bilateral choanal atresia in the other 50% (8). Female-male ratio showed 3:1 ratio. In patients with unilateral imperforation, right malformation was predominant in both sexes; bilateral imperforation was more frequent in females. Mixed imperforation (bone-membranous) was the most frequently observed type, followed by bone malformations.

The most common symptoms at diagnosis were respiratory failure − 18.04% (4), nasal obstruction − 33.3% (7), rhinorrhea − 28.5% (6), sinusitis − 4.76% (1), and otitis − 4.76% (1). Diagnosis was confirmed by computerized tomography of facial sinuses in 56.25% (9) and by nasofibroscopy in 31.25% (5).

Associated diseases were found in 10 patients presenting cleft lip and palate, Treacher-Collins syndrome, cardiac malformations, Rapp-Hodgkin syndrome, hydrocephaly, general craniofacial malformations and sensorineural hearing loss.

Surgery was performed via transpalatine access with stent placement in all patients, except for one patient who arrived at the service for restenosis treatment, and was submitted to this procedure via endoscopy without stent placement. Out of the patients submitted to initial surgical correction, only one presented restenosis. Age at first surgery was 1 month and 1 day up to 20 years (mean 10 years) and restenosis correction occurred on average at the age of 7 (first surgery at 15 days of age). Permanence of stent was 2 months on average.

Length of hospital stay was 5 days on the average, except for patients with cardiac malformation. Outpatient follow-up lasted 1 year on average (varying from 1 month to 2 years).

## DISCUSSION

Bilateral choanal atresia is a medical emergency, once newborns present single nasal breathing in the first 3 weeks of life^6^, whose maintenance of the air pathway through oral intubation or tracheotomy is required, until definitive correction is performed. Mean age for defect correction was 10 years, showing our service's peculiarity, since some of our patients presented cleft palates, yielding nasal airway maintenance and late surgical correction (patients without cleft palate were operated on within 10 to 20 days after diagnosis).

Unilateral choanal atresia rarely presents severe respiratory insufficiency leading to late diagnosis. Most frequent symptoms are nasal obstruction, rhinorrhea and sleep apnea, which may be easily taken for other nasal pathologies - for instance, allergic rhinitis, nasal polyposis and upper airways infectious processes. In our service, differently from the literature, mean age for treatment of unilateral atresia was 12.5 years^6^. We believe that this is due to lack of efficient health services in the country and delay in performing tests and referrals, leading to late diagnoses and, consequently, late treatment. Diagnostic suspicion occurred among patients with characteristic symptoms, after other causes of respiratory difficulty were eliminated. We initiated the physical examination with nostril inspection and posterior nasal intubation with nasogastric probe (NGP) of various gauges (we began with #8, decreasing to #4, should progression not be present). If the probe did not go through one nostril, or both of them, we chose computerized tomography of paranasal sinuses and nasofibroscopy. CT and nasofibroscopy are not always necessary for the diagnosis, although we considered them great tools to aid the surgical plan.

We used transpalatal technique in all patients, because we are skilled in this surgical technique and believe this is the best way for malformation exposure; however, literature reports that this technique is associated with longer surgical duration, larger bleeding, potential risk of bucconasal fistula, palatine dysfunction, facial growth disorder with cross bite and restenosis1., 2., 3.^,^^6^. Three patients had complications after transpalatal technique: 1 bleeding, 1 palate fistula and 1 restenosis case.

Endonasal techniques have been used due to increasing technical and surgical improvements. Presumably, these techniques present lower bleeding risks, shorter surgery time, although they may frequently present liquor fistulas and meningitis by fracture of ethmoidal crivous lamina, restenosis and incomplete resections1., 2., 3.^,^^6^. Only one patient was submitted to this technique for correction of perforation restenosis of bilateral membranous choanae along with turbinectomy, presenting postoperative bleeding and need of nasal packing.

The literature describing the importance of a stent in choanal imperforation surgery is restrict^10^. Most case series are small and frequently reflect the collective experience of several surgeons. In all our patients submitted to transpalatal technique, a stent was used during an average period of 2 months. Most authors agree with the use of a stent in transpalatal surgeries, although permanence time is still controversial, showing a range of 3–4 weeks^11^^,^^12^ to 2 days^13^. Despite the fact that the permanence time adopted by our service is longer than that discussed in the literature, none of the patients presented adverse events due to this issue. Schoem (2004), in his literature review, concluded that in endonasal technique, where tissue manipulation is lower, the use of stent may not be chosen. In our service, only one endonasal surgical correction was performed in a restenosis case referred from another service, where a stent had not been placed. So far, this patient has improved without any adverse events.

## CONCLUSION

Bilateral choanal imperforation is early diagnosed due to its exuberant symptomatology and high suspicion level by neonatology pediatricians. Unilateral imperforation is frequently treated as a pathology. Despite being uncommon, any patient with respiratory obstruction complaint should be carefully assessed, regardless of age and especially if he/she presents face anomalies, so that diagnosis and correction can be early conducted and ineffective and unnecessary treatments are avoided.

Despite the current development and improvement of endonasal technique and the small number of cases in our service, transpalatine technique, when well performed by a skilled surgeon, continues to be the safest way for correcting choanal imperforation and restenosis.

The use of stent for malformation correction was inconclusive, once our case series is restrict and did not present significant differences.
